# Reflections on the 150th anniversary of Naunyn–Schmiedeberg’s Archives of Pharmacology: past, challenges, and future

**DOI:** 10.1007/s00210-022-02321-4

**Published:** 2022-11-07

**Authors:** Yuichi Hattori, Roland Seifert

**Affiliations:** 1 Advanced Research Promotion Center, Health Sciences University of Hokkaido, Tobetsu, 061-0293 Japan; 2 Department of Molecular and Medical Pharmacology, Faculty of Medicine, University of Toyama, Toyama, 930-0194 Japan; 3 Institute of Pharmacology, Hannover Medical School, Carl-Neuberg-Str. 1, 30625 Hannover, Germany

## The past

*Naunyn-Schmeideberg’s Archives of Pharmacology* is the oldest pharmacology journal, founded in 1873 by Bernhard Naunyn, Oswald Schmiedeberg, and Edwin Klebs (Starke 1998; Seifert and Schirmer 2021; Dats et al. 2023). Oswald Schmiedeberg, a co-founder of the *Archives*, is generally recognized as the *pioneer of experimental pharmacology* and was the first to introduce the concept of pharmacodynamics and pharmacokinetics of a drug (Koch-Weser and Schechter 1978; Muscholl 1995; Starke 1998). Schmiedeberg received his education in pharmacology in Dorpart (Tartu) under the leadership of Rudolf Buchheim, the *founder of pharmacology*. The origins of the field of pharmacology are traced in a review article published in this issue (Philippu and Seifert 2023a). Another review in this issue analyzes the biographies of Schmiedeberg and several of his very successful students, the “Schmiedeberg school of pharmacology” (Philippu and Seifert, 2023b).

In his 46 years at the University of Strassburg (Strasbourg in French), 120 pupils and students from 20 different countries of the world came to study under his guidance. Schmiedeberg trained many students so successfully that they later became professors at other German universities and in several foreign countries including the UK, the USA, Italy, Austria, Switzerland, Norway, and Japan (Philippu and Seifert 2023b; Hattori et al. 2023). Schmiedeberg’s alumni and *Naunyn–Schmiedeberg’s Archives of Pharmacology* also played a major role in the highly successful establishment of pharmacology and the pharmaceutical industry in Japan as a global player (Hattori et al. 2023). From such circumstances, *Naunyn–Schmiedeberg’s Archives of Pharmacology* provided the best stage for pharmacologists from all over the world to publish their research in experimental pharmacology, German (and not yet English) being the language of science at that time (Starke 1998; Dats et al. 2023). Thus, *Naunyn-Schmideberg’s Archives of Pharmacology* quickly became one of the most highly respected scientific journals, exemplary in its standards (Koch-Weser and Schechter 1978). It is no exaggeration to state that *Naunyn–Schmiedeberg’s Archives of Pharmacology* was the journal that established the basis for modern experimental pharmacology.

## The challenges

Tragically, the closure for political reasons of the highly successful Pharmacology Department in Strassburg by the French government in 1918 after Germany’s defeat in World War I brought the Schmiedeberg era of pharmacology to an abrupt end (Philippu and Seifert 2023b). Even much more tragically, during the Nazi regime (“Nazi Deutsche or Nazi Germans”) (1933–1945), Germany and Austria suffered the exodus of many of their best pharmacologists to other countries including the UK and the USA (Löffelholz 2011). Other eminent pharmacologists died or were killed during this period. Among the victims of the Nazi regime were several Schmiedeberg alumni (Philippu and Seifert 2023b).

Attempts of the Nazi regime to restore an “Aryan Schmiedeberg school of pharmacology” in Strassburg from 1942 to 1944 were a complete failure (Philippu and Seifert 2023b). In addition, the destruction of most pharmacology departments in Germany during World War II had a decades-lasting negative impact on the scientific content and reputation of *Naunyn–Schmiedeberg’s Archives of Pharmacology*, bringing the journal to the brink of extinction (Starke 1998; Dats et al. 2023). The journal was only rescued by the very wise decision of Fred Lembeck and Ulrich Trendelenburg to switch the language of the journal from German to English in the 1970s. After the language switch, the journal strongly recovered and many important and highly cited papers were published in the journal (Dats et al. 2023). The period between 1975 and 2000 was probably the most impactful period of the journal on the development of modern pharmacology. It was also a period in which the pharmaceutical industry made major scientific contributions to the journal (Dats et al. 2023). However, a profound cultural shift in the academic research system in Germany encompassing universities and funding organizations alike happened at the turn of the Millenium. A new focus on (perceived) “Excellence” made it inevitable for pharmacologists (particularly junior researchers) in Germany to publish in journals with a higher impact factor than *Naunyn–Schmiedeberg’s Archives of Pharmacology* could offer, and the journal fell in disfavor with many German pharmacologists, again jeopardizing the existence of the journal (Zehetbauer et al. 2022; Dats et al. 2023b). Repeated urgent calls to the German pharmacology community to submit papers to the journal went largely unheard.

The Editors of *Naunyn–Schmiedeberg’s Archives of Pharmacology* (initially under the leadership of Martin C Michel and later Roland Seifert) continued to promote the journal by offering fair and constructive peer review (Seifert 2016). In fact, international publications have very well compensated for the massive loss of publications from Germany during the past 25 years (Dats et al. 2023). Ironically, the internationalization of publications in *Naunyn–Schmiedeberg’s Archives of Pharmacology* is a late fulfillment of the heritage of Oswald Schmiedeberg who started the process 150 years ago (Koch-Weser and Schechter 1978; Seifert and Philippu 2023b). *Naunyn–Schmiedeberg’s Archives of Pharmacology* survived even more challenges: In the 1970s–2000s, the journal had a strong and very successful focus on neurotransmitter pharmacology, Klaus Starke and Manfred Göthert being extraordinarily prolific contributors to the journal. Their retirements happened in parallel with the new focus in Germany on “Excellence” (Zehetbauer et al. 2022; Dats et al. 2023), and abruptly, the previously continuous flow of papers stopped. But over the past 25 years, this cessation of publications on neurotransmitter pharmacology was compensated by a rise in publications on immunopharmacology and tumor pharmacology (Dats et al., 2023). Many of the papers presently published in the journal deal with drugs isolated from plants, and the journal has implemented stringent rules for such papers (Merfort et al. 2017; Neumann and Seifert 2021).

The most recent challenge that jeopardized the journal occurred just 2 years ago when a massive attack of fake papers from so-called “paper mills” (criminal science publishing gangs) flooded the journal (Seifert 2021; Sabel and Seifert 2021). But through rigorous investigation of the fraud cases and implementation of stringent submission requirements, the journal quickly regained credibility and now serves as a role model for other scientific journals to fight fake publications.

## The future

For journal editors, it is not easy to precisely foresee the future of a journal and the content of future submissions. The legacy of Oswald Schmniedeberg is to have a broad and open-minded approach to pharmacology. His multiple research interests were extremely useful for the future development of different specialized research areas both in academia and the pharmaceutical industry (Philippu and Seifert 2023b). Schmiedeberg was nominated 18 times (!) for the Nobel Prize, but was never awarded the Prize; probably because his interests were too broad so that he did not follow just one research topic into the greatest detail (Pohar and Hansson 2020). In the tradition of Oswald Schmiedeberg, *Naunyn–Schmiedeberg’s Archives of Pharmacology* does not strive to publish research that will be the ultimate basis for a Nobel Prize, but the journal will remain open-minded for every solid research paper in the broad field of pharmacology. We explicitly welcome research from smaller fields of pharmacology with only a small (current) community. Experience has shown that it may take decades until the full scientific importance of research data, initially perceived as “marginal” by protagonists, is recognized (Seifert and Schirmer 2022). Open-mindedness was crucial for the successful transformation of *Naunyn–Schmiedeberg’s Archives of Pharmacology* from a “neurotransmitter” journal into a pharmacological journal that now covers many more research topics than 50 years ago (Dats et al. 2023). Scientific diversity is important for the future of the journal.

Genomics refers to the study of the entirety of an organism’s genes, called the genome, including interactions of those genes with each other and with the person’s environment. Genomics emerged in the 1980s and developed with the start of the Human Genome Project, which took 20 years and was completed in 2003, although more than a decade later genomics is still big business. The postgenomic era has made a drastic shift in research methodology. The biological methods for knowledge elucidation and pharmacological approaches to biomarker discovery have been rapidly changed in the postgenomic era. Postgenomics is transforming our understanding of disease and health. Indeed, differential expression of proteins in health and disease holds the key to early diagnosis and accelerating drug discovery. Our improved biomedical knowledge and understanding in the postgenomic era have led to advances in molecular and cellular biological research in the field of pharmacology. The science of pharmacology, which makes use of multi-disciplinary approach in looking into the reciprocal interactions of chemicals on biological systems, can place these advances in the appropriate physiological context of organ systems and intact animals.

Entering the postgenomic era, *Naunyn–Schmiedeberg’s Archives of Pharmacology* has faced a major turning point. *Naunyn–Schmiedeberg’s Archives of Pharmacology* has published many papers with the use of molecular biology techniques and genetically modified or transgenic animals. While the publication of the submissions which are descriptive and lack mechanistic novelty has been limited, *Naunyn–Schmiedeberg’s Archives of Pharmacology* has spared no effort to pick up papers covering research on the role of cellular signal transduction in pharmacology and toxicology.

In the future, *Naunyn–Schmiedeberg’s Archives of Pharmacology* will not only further develop the traditional areas of strength for the journal, including neuropharmacology, cardiovascular pharmacology, and more recently, immunopharmacology and tumor pharmacology (Dats et al. 2023) but also will undertake a mission of covering the futuristically attractive pharmacology areas such as those associated with an aging society. These areas include lifestyle diseases-related pharmacology, pharmacology of dementia such as Alzheimer’s disease, cancer pharmacotherapy, ocular pharmacology, and dental pharmacology. *Naunyn–Schmiedeberg’s Archives of Pharmacology* should expand and enhance the roles of editorials and reviews in the journal. Editorials are defined as an opinion or a view of a member of the editorial board or any senior or reputed faculty written in a journal and may be a commentary on a published article or a topic of current interest which has not been covered by the journal (Nundy et al. 2021). Editorials are expected to cover new developments in the field of pharmacology. We also welcome papers addressing current topis at the interface between pharmacology and society to highlight the overarching responsibility of our discipline (Zehetbauer et al. 2022). *Naunyn–Schmiedeberg’s Archives of Pharmacology* will continue to play a vital role in providing a place for communication of state-of-art-research on all aspects of pharmacology and its contribution to human health and to seek feedback from our authors and readers on different ways to further develop the journal and greatly advance the field of pharmacology.
